# Resorption of the Salts of Iron

**Published:** 1896-05

**Authors:** 


### Resorption of the Salts of Iron.

In the *Zeitsch. f. Physiolog. Chemie*, Hr. Woltering has a paper giving the results of an inquiry into the resorbability of iron salts into the system. He first repeated Kunkel's experiments, and found that when iron was given to mice, rabbits or dogs, by the mouth, it accumulated in the liver; that, in fact, the liver was possessed of the faculty of storing up iron. The question then arose as to the form in which it was thus stored up. The author found that one of the combinations was a nucleoproteid. In this form it was firmly associated and could not be discovered without destroying the substance. He found, also, by later experiment, that the accumulation of iron resulted directly from resorption, and not from any property of preventing waste.

A further question was, whether the organism could make use of the accumulated iron in case it required it for the production of hemoglobin, and the author's opinion is that it can. On withdrawing blood from animals fed with iron, and others as check animals, he found that the blood corpuscles did not fall so low in the former class as in the control animals. The blood returned to its normal state much more quickly and completely in the animals possessed of accumulated iron than in the others. It was very noticeable, also, that in the livers of the animals not fed with iron there was much less of it than in the others, and that in the iron animals the quantity was equal to, if not above, the normal amount. The author does not state how long a time is required for the organism to use up its surplus iron stored in this way.—*Berlin Cor. Med. Press and Circular.*

